# Novel In Situ Modification for Thermoplastic Starch Preparation based on *Arenga pinnata* Palm Starch

**DOI:** 10.3390/polym14224813

**Published:** 2022-11-09

**Authors:** Muhammad Ghozali, Yenny Meliana, Mochamad Chalid

**Affiliations:** 1 Green Polymer Technology Group, Department of Metallurgical and Material Engineering, Faculty of Engineering, Universitas Indonesia, Depok 16424, Indonesia; 2 Research Center for Chemistry, National Research and Innovation Agency (BRIN), Tangerang Selatan 15314, Indonesia

**Keywords:** thermoplastic starch, *Arenga pinnata*, modification, benzoyl peroxide, twin-screw extruder

## Abstract

Thermoplastic starch (TPS) has three main disadvantages, i.e., poor mechanical properties, low thermal stability and water sensibility. To overcome these disadvantages, TPS properties can be improved by starch modification, adding reinforcements and blending with other polymers. In this research, to prepare modified TPS, starch modification was carried out by in situ modification. The modified TPS was prepared by adding *Arenga pinnata* palm starch (APPS), glycerol and benzoyl peroxide simultaneously in the twin-screw extruder. Morphology analysis of TPS revealed that the starch granules were damaged and gelatinized in the extrusion process. No phase separation is observed in TPS, which exhibits that starch granules with and without benzoyl peroxide were uniformly dispersed in the matrix. The addition of benzoyl peroxide resulted in increased density of TPS from 1.37 to 1.39 g·cm^−3^, tensile strength from 7.19 to 8.61 MPa and viscosity from 2482.19 to 2604.60 Pa.s. However, it decreased the elongation at break of TPS from 33.95 to 30.16%, melt flow rate from 7.13 to 5.73 gr/10 min and glass transition temperature from 65 to 52 °C. In addition, the thermal analysis showed that the addition of benzoyl peroxide increased the thermal stability of TPS and extended the temperature range of thermal degradation.

## 1. Introduction

Thermoplastic starch (TPS) is considered one of the most promising alternatives to fossil-based ones for disposable packaging material applications [1], mainly because of its low price, biodegradability [2] and renewability [1]. TPS can be obtained from native starch granules found in numerous plants, such as rice, corn, wheat, cassava, potato [1], or sago [2]. However, the use of those starches will intersect with food sources. Therefore, other starch sources are needed in order to avoid the debate and criticism regarding the use of food sources [3]. Some fruit wastes can be extracted and considered as alternative sources of starch, including kiwifruit, pineapple stems, mango kernels, apple pulp, banana peel, litchi, tamarind, longan and loquat, annatto, jackfruit and avocado seeds [4]. However, the availability of these fruit wastes is limited. *Arenga pinnata* palm starch (APPS) is also considered an agro-industrial residue in the agricultural industry [5]. *A. pinnata* palm starch can be obtained from the core of an unproductive (in terms of sugar and fruit) *A. pinnata* palm tree’s trunk [6,7]. *A. pinnata* tree grows in more humid parts of subtropical and tropical areas [8]. It is widespread from South Asia to Southeast Asia and from the east of India [8] and Taiwan to Philippines, Indonesia, Papua New Guinea, India, North Australia, Malaysia, Thailand, Burma, Vietnam [3,7] and southwest of China [9]. Therefore, the abundant availability of APPS can be considered as a potential source of starch. A tree of *A. pinnata* can produce about 50–100 kg of APPS [8,10]. The starch content of APPS is approximately 10.5–36.7% [6] with 36.6 [6]–59.2% [8] amylose content. The density of APPS is around 1.54 g·cm^−3^ [3,11]. The gelatinization temperature of APPS was around 67 °C [6]. APPS was characterized by a C-type pattern crystalline structure [5,8]. In addition, *A. pinnata* palm starch has been used as a material for the preparation of TPS [5,9,10,11]. The density, tensile strength and elongation at break values of the TPS prepared from APPS are 1.41 g·cm^−3^, 4.8 MPa and 38.10%, respectively [5], while other studies reported a density value of TPS prepared from APPS of 1.40 g·cm^−3^ [11], tensile strength and elongation at break of 2.42 MPa and 8.03%, respectively [10].

TPS can be prepared by starch into TPS [12] under heat and shear in the presence of plasticizers [13,14]. The starch granules are destructurized, plasticized and melted, forming that has similar characteristics to that of thermoplastics [13]. Generally, the preparation process of TPS can be divided into two processes, the wet and dry process. The wet process is commonly used in the laboratory by solvent casting [15,16,17]. Solvent casting is a batch process, so time consuming [15], with factors of the need to evaporate large amounts of solvents [16], low in efficiency, high in cost [18] and not suitable for industrial production, while the dry process, usually via an extrusion process, is a continuous process, efficient and more suitable for industrial production. Extrusion is the most widely used for plastic films because of its advantages, such as simple production equipment, low investment, continuous production [15], easier handling, a broad range of processing conditions, good mixing [17] and easy scale-up [13,18,19]. Thus, the extrusion process is a promising approach to producing bio-based plastics [16] for industrial production.

However, compared to conventional plastics, TPS has three main drawbacks, i.e., low mechanical properties, low thermal stability as well as water and humidity sensibility [13,17,20]. To overcome these drawbacks, several solutions have been studied, such as chemical modification of starch, mixing with other polymers and incorporation of reinforcing materials [2,12,13,18,21]. Cellulose is one of promising reinforcing materials for enhancing mechanical properties of TPS [13,18]. Digestate sludge from an agricultural biogas plant is also considered as a promising reinforcing material for improving the mechanical properties of TPS biocomposites [22], while the modification of starch is generally modified physically, chemically, enzymatically or by combinations. Usually, chemical modification occurs via etherification, esterification and an oxidation process. Preparation of starches by introducing functional groups shows its helpfulness in reducing TPS’s hydrophilic performance and improving its compatibility. Starches are generally modified chemically to promote the hydrophobic, mechanical and thermal characteristics to increase TPS applications [21]. Oxidation is one chemical method for starch modification to obtain oxidized starch [23]. Oxidized starches also have the potential to be helpful in the preparation of biodegradable food packaging [21].

In the oxidized starch, the purpose of oxidation is to generate more functional groups, i.e., carbonyl and carboxyl, thereby increasing the functionality and reactivity of native starch. The preferred oxidants are hydrogen peroxide, sodium hypochlorite, potassium permanganate, chromic acid, nitrogen dioxide [21], ozone and sodium periodat [24]. The stage of oxidation in starch means the hydroxyl groups are oxidized to carbonyl groups first and then to carboxyl groups [24]. Oxidation reduced the relative crystallinity and viscosity of starches [21]. Starch oxidation improves moisture resistance with hydrophobic carbonyl groups, replacing the hydrophilic hydroxyl groups in starch, resulting in improved mechanical and thermal properties and lower humidity absorption compared to the TPS control film [17]. The use of oxidized starch improved the toughness, elongation at break, compatibility, thermal stability and rheological properties. However, it lowered the storage modulus and glass transition temperature of TPS [25].

Modifying starch by oxidation to obtain modified TPS has disadvantages, i.e., it requires time and cost and the process steps become longer because the starch is modified first and then the oxidized starch produced is used as raw material for TPS. The objective of this study is to overcome these disadvantages, in particular shortening the modified TPS preparation time. Therefore, in this study, starch modification was carried out via an in situ process simultaneously with the preparation of TPS with the extrusion process in the twin-screw extruder. 

## 2. Materials and Methods

### 2.1. Materials

In this research, *Arenga pinnata* palm starch (APPS) with an amylose content of 23.19% was obtained from the local industry located at Klaten, Central Java, Indonesia. Glycerol (CAS 56-61-5) and benzoyl peroxide (CAS 94-36-0) were purchased from Merck, Darmstadt, Germany.

### 2.2. Preparation of Thermoplastic Starch (TPS)

The preparation of thermoplastic starch (TPS) was conducted by a twin-screw extruder [2,12,13,14,15,16,17,18,19,25,26,27,28,29,30,31]. APPS (70%w), glycerol (30%w) and benzoyl peroxide (0.1 phr of the weight of APPS + glycerol) were mixed and stirred using a mixer until well distributed. Then the mixture was stored overnight (24 h) to diffuse the glycerol into the APPS granules completely. Furthermore, the mixture was fed manually into a co-rotating twin-screw extruder (Compounder ZK 16 T x 36 L/D, Collin, Germany) at a screw speed of 90 rpm with a temperature extruder barrel as the following profile 40/80/120/150/150/150/150/150 °C from zones 1–8. Then the extruded strips were cut into pellets (diameter 2–3 mm) by a pelletizer. In this study, when only glycerol is added, the thermoplastic starch is abbreviated as TPS and when glycerol and benzoyl peroxide are added, the thermoplastic starch is abbreviated as TPSB.

### 2.3. Fourier-Transform Infrared (FTIR)

The functional groups of the APPS, TPS and TPSB were obtained using Fourier-Transform Infrared Spectroscopy (Bruker Tensor II, Etlingen, Germany). Thus, 32 scans were recorded for each sample while it was set in attenuated total reflectance (ATR) mode with a diamond ATR crystal at a wave number range between 500 and 4000 cm^−1^.

### 2.4. Density Measurements

The density of the APPS, TPS and TPSB was measured (five replicates) in accordance with the ASTM D 792 standard at 23 °C and 50% relative humidity.

### 2.5. Scanning Electron Microscopy (SEM)

The surface morphology of APPS and the cross-section morphology of TPS and TPSB were observed using scanning electron microscopy (JEOL, JSM-IT200, Tokyo, Japan) at 3 kV for APPS and 10 kV for TPS and TPSB. After submersion in liquid nitrogen, the TPS and TPSB samples was broken. The surfaces had a thin gold layer applied to the sample before analysis.

### 2.6. X-ray Diffraction (XRD)

The crystallinity of APPS, TPS and TPSB was studied by X-ray diffractometer (Malvern Panalytical’s Aeris, Eindhoven, Netherlands). The X-ray diffraction pattern observed the 1.54 wavelength, 40 kV voltage and 15 mA filament emission. The radiation reflection of APPS, TPS and TPSB was measured at the 2θ angle of 5–50°.

### 2.7. Mechanical Properties

The mechanical properties of TPS and TPSB were determined using an electronic universal testing machine (UTM) (Shimadzu AG-X plus 50 kN, Kyoto, Japan), in accordance with ASTM D 638-14 standard, at 21 °C and relative humidity around 50%. Thermo Scientific Haake MiniJet Pro is used to prepare a dumbbell (dogbone) tensile test sample specimen according to ASTM D 638-14 type V. Five specimens of each type of TPS and TPSB film were calculated at tensile rates of 10 mm/min. Each sample was randomly measured three times at various locations using a digital thickness gauge (Preisser Digimet, Gammertingen, Germany) to determine the film thickness.

### 2.8. Rheological Properties

The rheological properties of TPS and TPSB were studied using the Melt Flow Index (MFI) Instrument Ceast Model 7026, in accordance with ASTM E 1238. Measurement of melt flow rate (MFR), viscosity and shear rate value was carried out at 190 °C with load 2.16 kg.

### 2.9. Differential Scanning Calorimeter (DSC)

The thermal properties of APPS, TPS and TPSB were characterized using differential scanning calorimetry (DSC) (DSC 4000, PerkinElmer, Waltham, MA, USA. DSC analysis was carried out with approximately 5 mg APPS, TPS and TPSB samples on a standard aluminum pan to determine the samples’ glass transition temperature (Tg). Each sample was heated until 200 °C with a 10 °C/min heating rate under a nitrogen atmosphere (flow rate = 20 mL/min).

### 2.10. Thermogravimetric Analysis (TGA)

The stability of APPS, TPS and TPSB was studied using a thermogravimetric analyzer (TGA 4000, PerkinElmer, Waltham, MA, USA). A crucible pan containing 5 mg of sample was heated from 25 °C to 600 °C at a rate of 10 °C/min. A flow of 20 mL/min was used to purge the nitrogen gas.

## 3. Results and Discussions

### 3.1. FTIR Analysis

**Figure 1:**
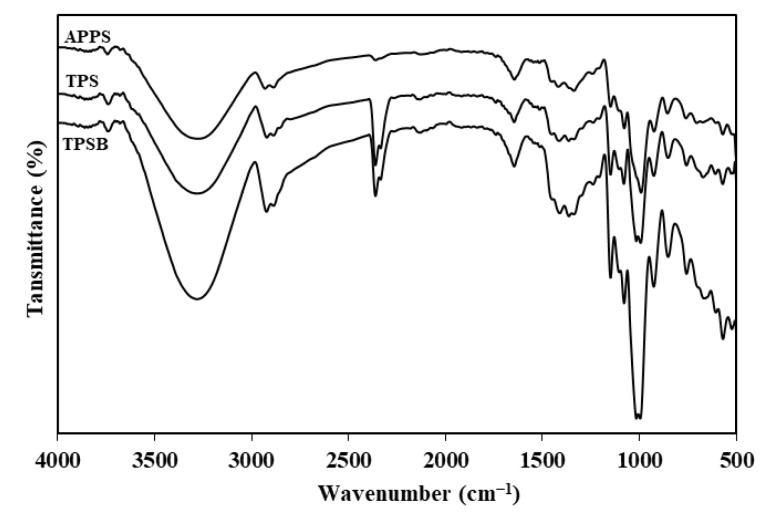
FTIR spectrum of APPS, TPS and TPSB.

The FTIR spectrum of APPS, TPS and TPSB are shown in Figure 1. The FTIR spectrum of APPS, TPS and TPSB shows absorption peaks at similar wave numbers. The absorption peak widened at a wave number of about 3265 cm^−1^, associated with the hydroxyl group from APPS and glycerol, while the absorption peak at a wave number of about 2921 and 2884 cm^−1^ was associated with the C–H group in starch [18,26]. In addition, the absorption peak at wave number 1645 cm^−1^ is the H–O–H vibration of water molecules in the amorphous region, which may be absorbed in the samples [16,18]. The absorption peaks around 1450–1330 cm^−1^ are associated with CH_2_ bending and wagging (out of plane bending) of CH_2_. The absorption peaks between 1500 cm^−1^ and 1200 cm^−1^ overlap each other between C–H stretching and O-H bending, making it difficult to distinguish the difference in the absorption peaks in this part of the spectrum [17]. Fuente et al., 2022 [16] reported the absorption peak at a wave number between 1200 cm^−1^ and 900 cm^−1^ is the vibration of a functional group of C–O, C–C and C–O–H. Similar absorption peaks of APPS were also reported by previous studies [5,8,10], while the FTIR spectrum result of TPS was verified by [5,10]. Zhang et al., 2013 [25] reported the hydroxyl groups of starch were changed to carbonyl and carboxyl groups during oxidation. Furthermore, these carbonyl and carboxyl groups of oxidized starch form strong hydrogen bonds with the hydroxyl groups on starch. The anhydroglucose ring of oxidized starch is still maintained, so its chemical structure looks like starch and TPS [25]. In addition, the lack of a significant difference in the absorption peaks of TPS and TPSB, possibly caused by the low concentration of benzoyl peroxide used, causes changes in the functional groups of starch molecules that are not drastic enough to be identified by this technique.

### 3.2. Physical Properties

**Figure 2:**
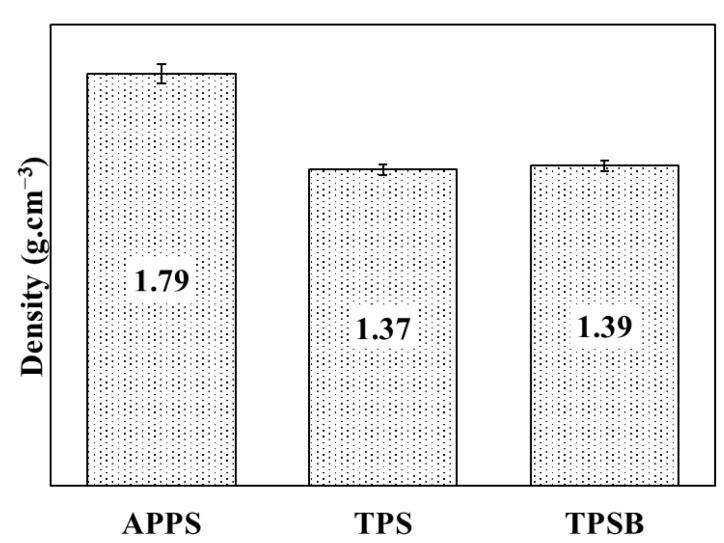
Density of APPS, TPS and TPSB.

The density of APPS, TPS and TPSB is shown in Figure 2. The density values of APPS, TPS and TPSB were 1.79, 1.37 and 1.39 g·cm^−3^, respectively. The presence of glycerol as a plasticizer destroys and weakens the inter- and intra-molecular hydrogen bonding between starch molecules, thereby increasing the free volume and mobility between molecular chains and decreasing the density of TPS [5,11,25]. Previous solvent-casting methods reported that TPS’s density values varied from 1.40 g·cm^−3^ [11] to 1.41 g·cm^−3^ [5]. Compared to the solvent-casting method, the density of TPS in this research was lower, which proved that the extrusion method could potentially increase the interaction between the glycerol and APPS, leading to an increase in free volume and mobility between molecular chains. The use of benzoyl peroxide as an oxidizing agent on in situ modified TPS preparation increases the TPS density, although not very significantly, from 1.37 g·cm^−3^ to 1.39 g·cm^−3^. The higher density value of TPSB compared to TPS is, presumably, because the TPSB molecules are arranged more neatly and orderly so that the density value is greater than the TPS density value. The extrusion process is expected to produce thermoplastic materials with more neatly and consistently ordered molecules [16]. In addition, during the extrusion process, oxidation of the starch molecule may lead to the formation of carbonyl or carboxyl groups. The carbonyl groups that may be formed can form strong hydrogen bonds with the hydroxyl groups of starch, resulting in a stiffer film and increase in density. However, because the use of benzoyl peroxide is very small, the result changes are also not significant. The insignificant changes were also confirmed by the absence of new functional groups in the results of the FTIR analysis.

### 3.3. Morphology

**Figure 3:**
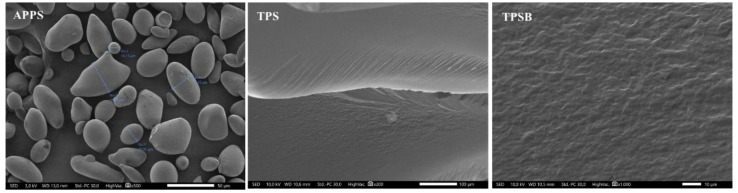
Surface morphology of APPS and cross-section morphology of TPS and TPSB.

The morphology of APPS, TPS and TPSB was examined by SEM. The samples were fractured in liquid nitrogen before testing. Figure 3 shows the surface morphology of APPS and the cross-section morphology of TPS and TPSB. Figure 3 shows that the APPS used in this study is granular and has inhomogeneous shapes and sizes. Some are spherical, oval and irregular, with diameters between 13 and 52 mm. Similar results on the morphology of APPS were also reported [8]. The TPS cross-section surface is rather smooth, meaning the APPS granules changed phase during the process in the extruder. The starch granules were physically broken into small fragments and melted due to the continuous interaction of the plasticizer, heat and shear rate in the twin-screw extruder, which resulted in smoother morphology and the disappearance of the granular structure of starch [25]. In addition, no phase separation is observed in TPSB, which exhibits starch granules with and without benzoyl peroxide that are uniformly dispersed in the matrix. Further, it indicates a good compatibility and plasticization process of starch with glycerol in the twin-screw extruder [15,25].

### 3.4. Crystallinity

**Figure 4:**
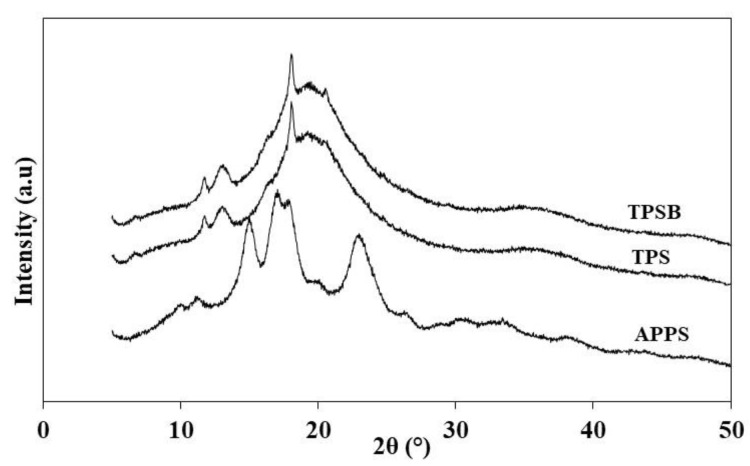
XRD pattern of APPS, TPS and TPSB.

The X-ray diffraction pattern of APPS, TPS and TPSB shows the semi-crystalline (presence of amorphous and crystalline) characteristic, as can be observed in Figure 4. In Figure 4, APPS shows diffraction peaks with high intensity at 2θ of 15.1°, 17.2°, 18.0° and 23.3°, which indicates that the characteristic palm starch has a C-type pattern crystalline structure. The XRD result of APPS was confirmed by previous studies [5,6,8]. The change in the crystalline structure of APPS after processing with glycerol and benzoyl peroxide is clearly visible in the X-ray diffraction pattern. The TPS and TPSB have similar diffraction patterns. The TPS and TPSB diffraction patterns show diffraction peaks at 2θ of 11.9°; 13.3° and 18.0° and did not show any diffraction peak crystallinity type C of APPS, proving that the initial APPS granules were gelatinized during the thermoplasticization process in the twin-screw extruder. In addition, TPS and TPSB show a broad hump diffraction peak pattern at 2θ 19°. The broad hump diffraction peak pattern at 19° is a characteristic of completely amorphous material [13]. This indicates that TPS and TPSB were not completely amorphous. Amorphous regions are caused by the disruption of the double-helix conformations of the starch due to the starch gelatinization, while the crystalline regions were formed by the recrystallization, favored by the formation of microcrystalline connections due to the presence of glycerol [16].

In addition, at TPS and TPSB, new diffraction peaks also appeared at 2θ 13.39°, 18.13° and 20.68°. These peaks are characteristic crystallinities type VH, which is formed during thermo-mechanical processing in the twin-screw extruder [13]. This proves that there was a change in the crystallinity structure of APPS to TPS and TPSB, from type C to VH. The diffraction peak at 2θ 20.68° in TPSB has a higher intensity compared to TPS. This kind of feature is caused by the presence of benzoyl peroxide during the extrusion process at TPSB. After the extrusion process, types of crystallinnity can be distinguished in TPSs: (i) residual crystallinity, native A, B or C crystallinity, which incompletely destroy and melt starch granules during the process and (ii) processing-induced crystallinity, V_H_, V_A_ or E_H_ crystallinity, which is formed during the thermo-mechanical process [13]. TPS shows process-induced crystallinity due to the hot-processing process, caused by the crystallinity in the starch chain, compounded with plasticizer and water into a single-helix structure. This degree of crystallinity was induced by hot processing, attributed to a strong interaction among the hydroxyl groups of the starch molecular chain, which was replaced by a hydrogen bond formed between the starch and the plasticizer [18]. The presence of glycerol as a plasticizer increases the mobility of the molecular chains and causes crystallization [32]. The extrusion process resulted in damage to the crystal structure of the native starch, as shown by the difference in diffraction patterns between APPS, TPS and TPSB. However, the glycerol induces plasticization of the starch chains during extrusion and will recrystallize the starch [1].

### 3.5. Mechanical Properties

**Figure 5:**
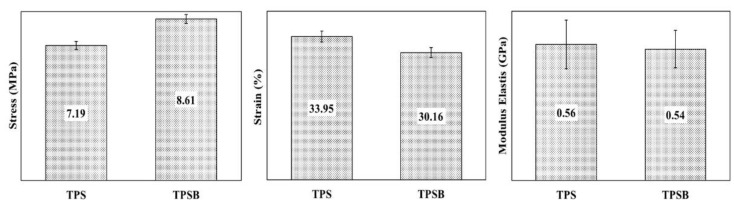
Mechanical properties of TPS and TPSB.

The mechanical properties in the form of tensile strength, elongation and elastic modulus of TPS and TPSB are shown in Figure 5. In Figure 5, the tensile strength, elongation at break and elastic modulus of TPS were 7.19 MPa, 33.95% and 0.56 Gpa and TPSB were 8.61 MPa, 30.16% and 0.54 Gpa, respectively. However, previous research on TPS preparation from APPS by solvent casting reported the tensile strength and elongation at break of the TPS films from APPS of 4.8 MPa and 38.10% [5] and 2.42 MPa and 8.03% [10], respectively. The tensile strength value of TPS obtained in this study was higher, which proved that the use of the extrusion method in the preparation of TPS could further increase the interaction between the plasticizer and APPS compared to the solvent-casting method. A strong correlation between the processing method applied and the mechanical properties of TPS was reported in the literature [5]. Further, Zhang et al., 2013 reported tensile strength of TPS from oxidized corn starch of 1.0–2.1 MPa and elongation at break of 131.7–170.2% [25]. These results are also different from the mechanical properties produced in this study. This might have occured due to the different types of starch used. In addition, the increase in tensile strength proves that the addition of benzoyl peroxide can increase the tensile strength of TPS. During the extrusion process, the benzoyl peroxide as an oxidizing agent resulted in the oxidation of the starch molecule, which was expected to cause the formation of a carbonyl or carboxyl group. Either carbonyl or carboxyl group are able to form strong hydrogen bonds with the hydroxyl groups of starch, resulting in a stiffer film and increasing the tensile strength of TPSB [16]. However, the addition of an oxidizing agent will also result in a decrease in the elongation at break of TPSB, from 33.95% to 30.16% and elastic modulus from 0.56 Gpa to 0.54 GPa.

The stiffer TPSB results in limited mobility of the molecular chain in TPSB, thereby reducing flexibility, which results in a decrease in elongation at break. In addition, in the extrusion process, depolymerization also occurs, which facilitates the tendency of molecular reassociation, with a greater potential for interaction [16]. Therefore, the new polymer matrix formed has different interactions between starch molecules and glycerol, so as to produce stronger bioplastics. In addition, the extrusion process can also support the alignment of the chain in the direction of flow, which results in a more flexible material. Therefore, extrusion is expected to produce thermoplastic materials whose molecules are more neatly and orderly arranged so as to increase the tensile strength of thermoplastic materials [16]. 

### 3.6. Rheological Properties

**Figure 6:**
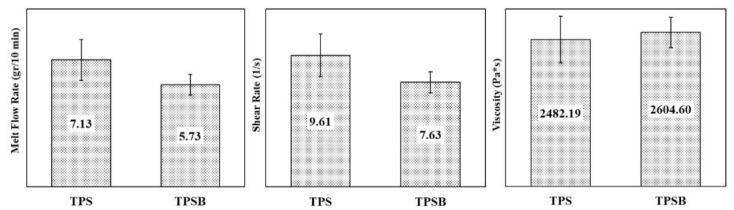
MFR, shear rate and viscosity TPS and TPSB.

The melt flow rate (MFR), shear rate and viscosity of TPS and TPSB are shown in Figure 6. The presence of benzoyl peroxide in TPS showed a decrease in the MFR value from 7.13 gr/10 min to 5.73 gr/10 min. The benzoyl peroxide, as an oxidizing agent, resulted in the oxidation of the starch molecule, which was expected to cause the formation of a carbonyl or carboxyl group. Either carbonyl or carboxyl group are able to form strong hydrogen bonds with the hydroxyl groups of starch [16]. These strong hydrogen bonds with the hydroxyl groups of starch reduce the molecular mobility of polymers, resulting in a stiffer film of TPSB film and inhibiting the flow rate of the TPSB, thereby reducing the amount of TPSB material that comes out of the MFI instrument barrel, resulting in a decrease in the MFR value of TPSB. The presence of benzoyl peroxide, which makes TPSB stiffer and harder to flow, also causes an increase in viscosity from 2482.19 Pa.s to 2604.60 Pa.s and a decrease in shear rate from 9.61 s^−1^ to 7.63 s^−1^.

The strong hydrogen bonds between oxidized starch with hydroxyl groups of starch will result in a decrease in chain mobility of TPSB, an increase in stiffness, an increase in viscosity and a decrease in shear rate, thereby reducing the melt flow rate of TPSB. The relationship between viscosity and shear rate has also been revealed [25]: when the flow resistance is reduced, the shear rate will increase and the viscosity will decrease, indicating that the melted starch mixture extruded behaves like a pseudoplastic liquid. Furthermore, as the shear rate increases, the chain entanglement in starch decreases, which leads to a weakening of the inter- and intra-molecular interactions between starches, thereby reducing the flow resistance. Therefore, a compatible and well-dispersed mixture can be characterized by increasing the shear rate and decreasing the viscosity [25].

### 3.7. Thermal Properties

**Figure 7:**
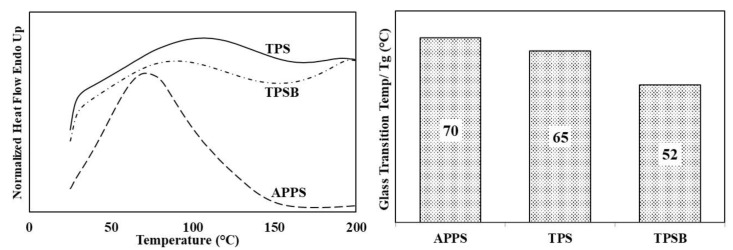
DSC curve and glass trasnsition temperature of APPS, TPS and TPSB.

The DSC curve and the glass transition temperature (Tg) APPS, TPS and TPSB are shown in Figure 7 and Table 1. It can be seen that the peak gelatinization temperature of APPS was 70 °C, close to a previously reported peak temperature of gelatinization of APPS of 67 °C [6]. However, it was lower than the results in the literature, which reported that the gelatinization temperature of APPS was around 98 °C [8]. In addition, Tg values of TPS and TPSB were 65 °C and 52 °C, respectively. The Tg value of TPS was lower than the gelatinization temperature of APPS. The decrease in the Tg value was related to the structure of the APPS granules being destroyed by glycerol during the extrusion process at high temperatures [18]. The plasticization process by glycerol reduces and exchanges the inter- and intra-molecular bonds between starch with a starch–glycerol hydrogen bond, increases free volume and increases chain mobility and intermolecular spacing, thereby improving the flexibility of TPS, thus, leading to a reduction in Tg [3,10]. The use of benzoyl peroxide in TPS preparation further reduces the Tg value. These show that the chain mobilities and flexibilities of TPSB are enhanced due to the fact that the addition of oxidized starch interrupts the hydrogen bonds between starch chains. Similar results were also reported by a previous study, where the oxidized starch had a stronger interaction between oxidized starch and starch chains, compared to interaction between starch, which leads to an increase in the mobility of starch chains [25].

**Figure 8:**
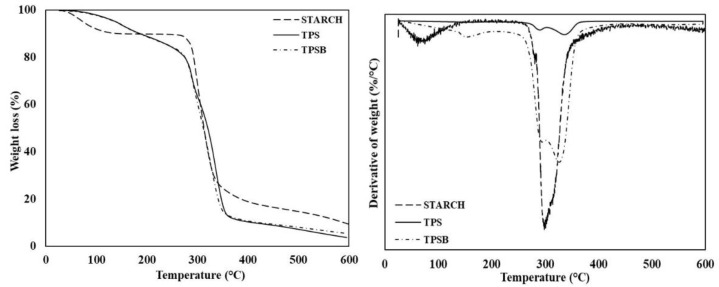
Thermal stability of APPS, TPS and TPSB.

Thermal stability of APPS, TPS and TPSB was studied by TGA. Figure 8 shows the thermal stability curve of APPS, TPS and TPSB. The APPS showed two stages of thermal degradation. The initial stage of thermal degradation occurred at temperatures up to 150 °C, with the peak of thermal degradation occurring at 70 °C. This initial thermal degradation was correlated to the evaporation of water. A similar TGA pattern of APPS was reported in the literature [10]. Thermal degradation in the second stage, as the main thermal degradation, occurred in a temperature range of 260–400 °C, with the peak of main thermal degradation occurring at 300 °C. This main thermal degradation was also correlated to the dehydration of starch molecules to form glucose. Figure 8 and Table 1 also show that the final APPS residue at 600 °C was about 9.36%, associated with the partial carbonization of starch. These results are similar to the previous study’s findings [18].

The thermal degradation curve of TPS also has two stages of thermal degradation, which are similar to those of APPS. The initial thermal degradation is also related to the evaporation of water. However, the initial thermal degradation of TPS has a different pattern with APPS and the thermal degradation temperature range in the early stages is higher, up to 180 °C. In addition to that, the peak of thermal degradation in the early stages of TPS occurs at a temperature of 150 °C. The main thermal degradation of TPS occurs in a temperature range of 260–400 °C and shows two main stages of thermal degradation. The first major thermal degradation is at a temperature of 260–310 °C, with a peak of thermal degradation at 290 °C, associated with the decomposition of the glycerol-rich phase, while the second major thermal degradation occurs in a temperature range of 310–400 °C, with a peak thermal degradation at a temperature of 330 °C, associated with the degradation of amylose and amylopectin starch. This result has good accordance with a previous study [15,16,26]. The presence of glycerol causes the peak temperature of TPS thermal degradation to shift towards higher temperatures. As a result, the glycerol in starch provides an advantage in thermal stability by increasing the mobility of the molecular chains due to the plasticization process, thereby increasing the fluidity of the material and delaying the decomposition of the material caused by the process at high temperatures [15]. The thermal degradation of TPSB shows a pattern of thermal degradation that is similar to the pattern of thermal degradation of TPS. However, the use of benzoyl peroxide in the preparation of TPSB increased the thermal resistance and extended the thermal degradation temperature range of TPSB when compared to TPS. This is evidenced by the mass remaining at a temperature of 600 °C, i.e., TPSB is 5.51%, while TPS is 5.47% (Table 1). This is presumably because oxidized starch increases the interaction of glycerol to starch by hydrogen bonding, thus, making them more difficult to evaporate during processing. This proves that oxidized starch can improve the thermal stability of TPS, which means that the addition of oxidized starch prevents the degradation of starch-based materials at processing temperatures [25].

## 4. Conclusions

In this research, in situ modification for TPS preparation based on *Arenga pinnata* palm starch was successfully carried out. Modified TPS was prepared by adding palm starch, glycerol and benzoyl peroxide simultaneously with the twin-screw extruder. Morphology analysis of TPS showed that the starch granules were damaged and gelatinized in the extrusion process. No phase separation is observed in TPSB, which exhibits that starch granules with and without benzoyl peroxide are uniformly dispersed in the matrix. The use of benzoyl peroxide in the preparation of TPS increases the density, tensile strength, viscosity and thermal stability as well as extending the thermal degradation temperature range of TPS. However, it reduces elongation at break, elastic modulus, melt flow rate, shear rate and glass transition temperature. The results of this study are expected to provide insight into the potential of *A. pinnata* as a new starch source for the development of biodegradable materials. In addition, in situ modification for the preparation of modified TPS with the extrusion process in the twin-screw extruder can reduce TPS preparation time because it only requires one preparation stage, meaning there is no need to modify the starch first. The characteristics of modified TPS produced in this study are expected to contribute to the further development of biodegradable materials.

## Figures and Tables

**Table 1:** Thermal properties of APPS, TPS and TPSB.

Sample	APPS	TPS	TPSB
Gelatinization temperature (°C)	70	-	-
Glass transition temperature (°C)	-	65	52
Residual mass at 600 °C (%)	9.36	5.47	5.51

## Data Availability

The data presented in this study are available on request from the corresponding author.
